# PAK inhibitor FRAX486 decreases the metastatic potential of triple-negative breast cancer cells by blocking autophagy

**DOI:** 10.1038/s41416-023-02523-4

**Published:** 2023-12-18

**Authors:** Liang Lyu, Haiyan Li, Kefeng Lu, Shu Jiang, Huihui Li

**Affiliations:** 1 Department of Neurosurgery, State Key Laboratory of Biotherapy, West China Hospital, Sichuan University, Chengdu, 610041 China; 2 West China Second University Hospital, Sichuan University, Chengdu, 610041 China

**Keywords:** Breast cancer, Macroautophagy

## Abstract

**Background:**

Triple-negative breast cancer (TNBC) is a unique breast cancer subtype with a high risk of metastasis and recurrence and a poor prognosis. Epithelial-mesenchymal transition (EMT) endows epithelial cells with the ability to move to distant sites, which is essential for the metastasis of TNBC to organs, including the lung. Autophagy, an intracellular degradation process that involves formation of double-layered lipid autophagosomes that transport cytosolic cargoes into lysosomes via autophagosome–lysosome fusion, is involved in various diseases, including cancer and neurodegenerative, metabolic, cardiovascular, and infectious diseases. The relationship between autophagy and cancer has become relatively clear. However, research on pharmacological drugs that block cancer EMT by targeting autophagy is still in the initial stages. Therefore, the re-evaluation of old drugs for their potential in blocking both autophagy and EMT was conducted.

**Methods:**

More than 2000 small molecule chemicals were screened for dual autophagy/EMT inhibitors, and FRAX486 was identified as the best candidate inhibitor of autophagy/EMT. The functions of FRAX486 in TNBC metastasis were detected by CCK-8, migration and wound healing assays. The effects of FRAX486 on autophagy and its target PAK2 were determined by immunoblotting, immunofluorescence, immunoprecipitation analysis and transmission electron microscopy. The findings were validated in mouse models.

**Results:**

Here, we report that FRAX486, a potent P21-activated kinase 2 (PAK2) inhibitor, facilitates TNBC suppression both in vitro and in vivo by blocking autophagy. Mechanistically, FRAX486 inhibits autophagy in TNBC cells by targeting PAK2, leading to the ubiquitination and proteasomal degradation of STX17, which mediates autophagosome–lysosome fusion. The inhibition of autophagy by FRAX486 causes upregulation of the epithelial marker protein E-cadherin and thus suppresses the migration and metastasis of TNBC cells.

**Conclusions:**

The effects of FRAX486 on TNBC metastasis suppression were verified to be dependent on PAK2 and autophagy inhibition. Together, our results provide a molecular basis for the application of FRAX486 as a potential treatment for inhibiting the metastasis of TNBC.

## Introduction

Breast cancer (BC) has become the most frequently diagnosed cancer type and the fifth-ranked cause of cancer-related death in the world [1, 2]. BC constitutes 22.9% of all cancers in females and is highly heterogeneous at the histological and molecular levels [2]. Triple-negative breast cancer (TNBC) is the most advanced breast cancer subtype with a high risk for metastasis and recurrence and a poor prognosis with current therapeutic modalities [3, 4]. Due to the lack of oestrogen receptor (ER), progesterone receptor (PR) and human epidermal growth factor receptor 2 (HER2) expression [5], endocrine therapy, which achieves a relatively satisfactory prognosis in ER/PR- or HER2-positive BC [6], is not suitable for TNBC [5, 7]. More importantly, advanced TNBC is incurable due to its high risk of recurrence and metastasis [8, 9]. Even though decades of research on TNBC have been fruitful, therapeutic options in TNBC are still one of the major challenges and unmet needs in the field [10]. Therefore, exploring the cellular and molecular mechanisms of the metastasis and recurrence of TNBC is crucial for developing effective drugs.

Autophagy is an intracellular catabolic process for the degradation of unwanted or dysfunctional materials in the cytosol [11, 12]. Autophagy dysfunction is associated with various diseases, such as cancer, metabolic diseases, neurodegenerative disorders, cardiovascular diseases, and infectious diseases. Exploring the implication of autophagy in these diseases could provide real benefits in patients and could be crucial for improving clinical diagnosis and treatment.

Autophagy is essential for maintaining homoeostasis in cancer cells [13] and has been considered a double-edged sword in cancer [14]. In the early stage of cancer development, the activation of autophagy is an inhibitory factor for metastasis-related signalling pathways [15, 16]. However, in the late stage of cancer progression, autophagy acts as a survival mechanism to sustain tumour cell viability, promotes the migration and invasion abilities of cancer cells, and consequently facilitates cancer metastasis [14, 17]. The relationship between autophagy and cancer has become relatively clear. However, to eliminate cancer, reverse drug resistance and prevent recurrence, more efforts to understand the role of autophagy in cancer development still need to be made.

Epithelial-mesenchymal transition (EMT) endows epithelial cells with the ability to move to distant sites [18]. The EMT process remodels the cytoskeleton and cell junctions of cancer cells and promotes the transformation from epithelial to mesenchymal morphology of cancer cells [19]. Therefore, EMT is a crucial cellular process that facilitates cancer invasion and metastasis in malignancies. In addition, EMT also induces a mesenchymal cell phenotype, which is related to higher chemoresistance risk and a worse prognosis [19]. Of note, the crosstalk between autophagy and EMT is complex [14, 20, 21]. Increased autophagic flux may support the survival of potential metastasis of cancer cells in renal cell carcinoma [20]. Other evidence has also revealed the repression of EMT by autophagy activation [21]. Therefore, inhibition of EMT by targeting autophagy might be a novel strategy for anticancer therapy. However, pharmacological drugs that cure cancer EMT by targeting autophagy are still in their infancy. Therefore, the re-evaluation of old drugs for their dual autophagy/EMT targeting potential was conducted.

Considering the pro-cancer effects of autophagy and EMT in advanced TNBC, dual autophagy/EMT inhibition is a promising approach for TNBC treatment. In our work here, the dual autophagy/EMT inhibitor FRAX486, a type I p21-activated kinase (PAK) inhibitor [22–24], was identified. We verified the specific and effective inhibitory effects of FRAX486 on the metastasis of TNBC cells. FRAX486 inhibits the downstream autophagosome–lysosome fusion of autophagy processes by promoting the degradation of the fusion factor STX17 in TNBC cells, which is dependent on the inhibition of PAK2. Autophagy repression by FRAX486 blocks the autophagic degradation of the epithelial factor E-cadherin and thus inhibits the in vitro and in vivo lung migration of TNBC cells.

## Results

### FRAX486 inhibits TNBC invasion and metastasis in vitro and in vivo

**Fig. 1:**
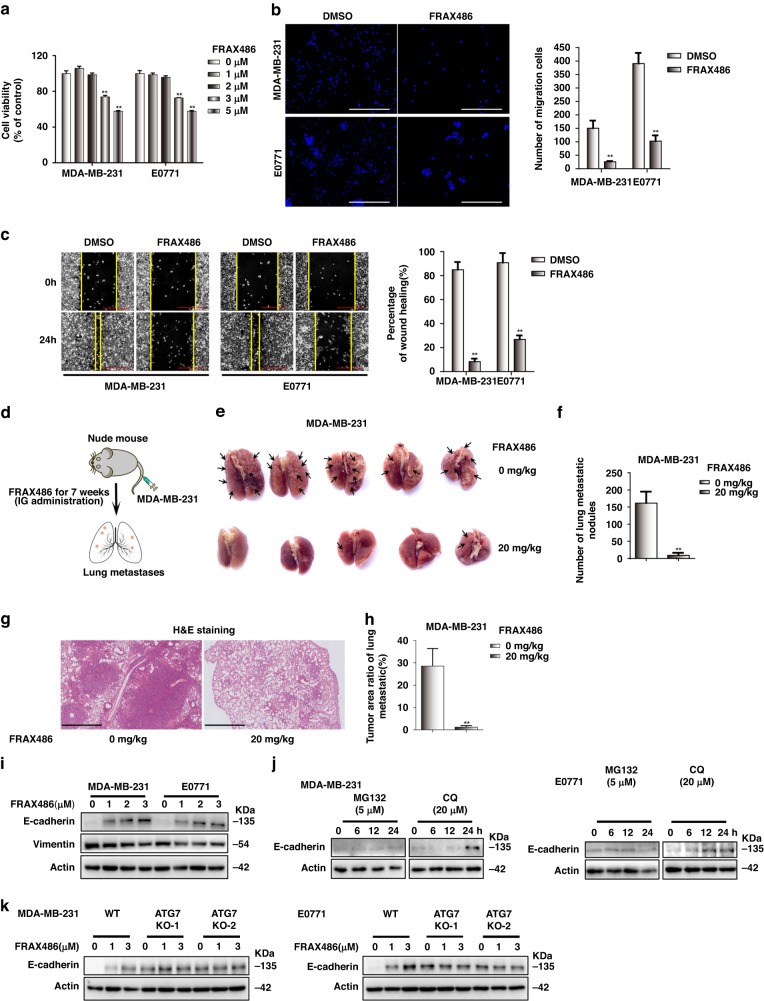
FRAX486 inhibits metastasis and the autophagic degradation of E-cadherin in TNBC cells. **a** CCK-8 assay was measured in MDA-MB-231 and E0771 cells treated with the appointed concentrations of FRAX486 for 72 h. **b** FRAX486 inhibited migration of TBNC cells determined by migration assay. Transwell chambers images of cell migration (left), number of migration cells (right). Scale bar: 500 μm. **c** FRAX486 inhibited migration of TBNC cells determined by wound healing assay. Wound healing images (left), wound healing rate (right). Scale bar: 500 μm. **d** Nude mice (*n* = 5 per group) were injected with 2 × 10^6^ WT, ATG7 knockout, PAK2 knockdown MDA-MB-231 cells via tail vein, and treated with vehicle and 20 mg/kg FRAX486 by gavage for 5 days each week. After 7 weeks’ treatment, lung tissues were collected, and metastatic nodules were quantified. **e** Lung metastasis of MDA-MB-231 cells in Nude mice (*n* = 5 per group). **f** The number of metastatic nodules in the lung. **g** Pathologic analysis of lung tissues by HE staining. Scale bar: 500 μm. **h** Tumour area ratio of lung metastatic. **i** Immunoblots analysis of E-cadherin and Vimentin in TNBC cells treated with the indicated concentrations of FRAX486 for 24 h. **j** The effect of MG132 or CQ on E-cadherin degradation in TNBC MDA-MB-231 and E0771 cells. **k** Effect of ATG7 knockout and FRAX486 on E-cadherin protein levels in TNBC MDA-MB-231 and E0771 cells. The data shown as mean ± SD and represent 3 independent experiments, and *p < 0.05; **p < 0.01 compared with DMSO treatment only.

We screened more than 2000 small molecule chemicals for dual autophagy/EMT inhibitors and identified that FRAX486 presents the most competent capability in autophagy/EMT inhibition (representative data are shown in Supplementary Fig. S1a, b). We verified the effects of FRAX486 on TNBC invasion and metastasis (Fig. 1). A dose of 2 μM FRAX486 was suitable for further examinations with limited influences on the viability of TNBC MDA-MB-231 and E0771 cells (Fig. 1a). In migration and wound healing assays, FRAX486 significantly inhibited cell migration (Fig. 1b, c). Next, cancer metastatic models generated by caudal vein injection of MDA-MB-231 cells were used to verify the in vivo effects of FRAX486 on TNBC cell migration and invasion (Fig. 1d). Compared with that in the control group, the number of lung metastatic nodules of TNBC MDA-MB-231 cells was dramatically decreased in the FRAX486 treatment group (Fig. 1e, f), while the weights of nude mice were not obviously affected by FRAX486 (Supplementary Fig. S2a). HE staining was carried out to confirm cancer metastasis in mouse models. The size and number of metastatic nodules were dramatically reduced by FRAX486 treatment (Fig. 1g, h and Supplementary Fig. S2b).

These data revealed that FRAX486 significantly inhibits TNBC migration and metastasis both in vitro and in vivo.

### FRAX486 inhibits the autophagic degradation of the epithelial protein E-cadherin in TNBC cells

Analysis of the protein levels of the epithelial protein E-cadherin and mesenchymal protein Vimentin revealed upregulation of E-cadherin and downregulation of Vimentin by FRAX486 treatment in TNBC MDA-MB-231 and E0771 cells (Fig. 1i and Supplementary Fig. S3a). Increased protein levels of E-cadherin could be a result of activation of gene transcription or inhibition of protein degradation. The mRNA level of CDH1 (E-cadherin-encoding gene) was slightly affected or not affected by FRAX486 in MDA-MB-231 and E0771 cells (Supplementary Fig. S3b). Thus, the increased protein levels of E-cadherin were speculated to result from the inhibition of degradation. MG132 (a proteasome inhibitor) did not increase the protein level of E-cadherin in TNBC cells; in contrast, chloroquine (CQ, an autophagy inhibitor) treatment caused the upregulation of E-cadherin at the protein level (Fig. 1g and Supplementary Fig. S3c). These data indicated that E-cadherin might be degraded via the autophagic pathway. Thus, TNBC cells with knockout (KO) of ATG7 (an autophagy gene) were constructed (Supplementary Fig. S3d). The results revealed that autophagy blockade by ATG7 KO increased E-cadherin protein levels (Fig. 1k and Supplementary Fig. S3e). More importantly, FRAX486 increased E-cadherin levels in wild-type (WT) TNBC cells, whereas it did not increase E-cadherin protein levels in ATG7 KO TNBC cells (Fig. 1k and Supplementary Fig. S3e).

Together, these results demonstrated that FRAX486 inhibits the autophagic degradation of the epithelial protein E-cadherin in TNBC cells, which is a potential mechanism by which FRAX486 represses TNBC migration and metastasis.

### FRAX486 represses TNBC migration via autophagy inhibition

Recent studies have revealed that activation of the autophagic degradation of E-cadherin promotes tumour EMT and thus the migration and metastasis of lung squamous cell carcinoma, melanoma and liver cancer cells [25–28]. Since the above results showed that FRAX486 represses TNBC invasion and metastasis and inhibits autophagy (suggested by the blockage of autophagic degradation of E-cadherin), we tried to clarify whether the effect of FRAX486 repression of TNBC invasion and metastasis occurs via autophagy inhibition.

**Fig. 2:**
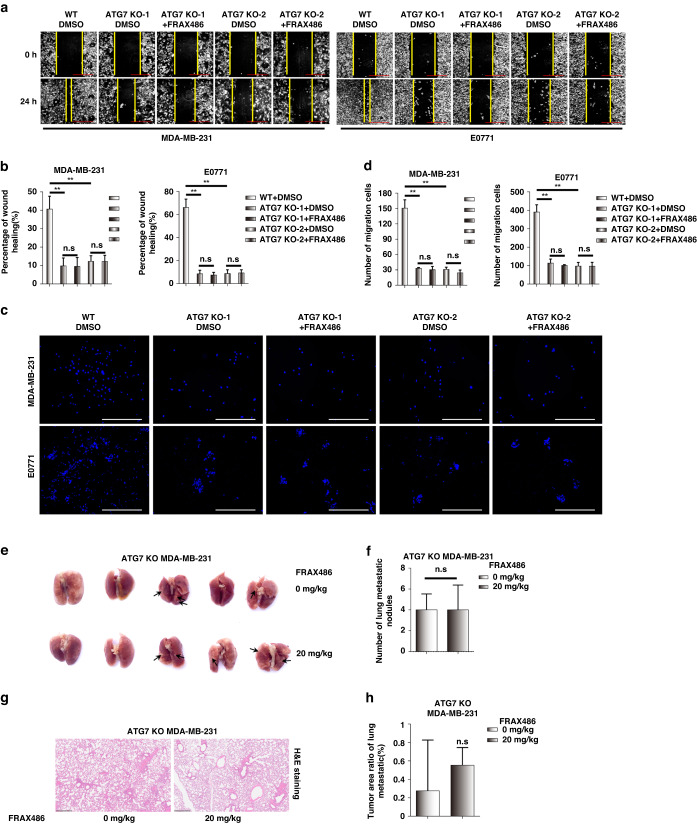
FRAX486 suppresses TNBC migration and metastasis via autophagy inhibition. **a**, **b** Wound healing assay detected the migration in WT and ATG7 knockout TNBC MDA-MB-231 and E0771 cells treated with FRAX486. **c**, **d** Migration assay detected the migration in WT and ATG7 knockout TNBC cells treated with or without FRAX486. **e** Lung metastasis of ATG7 knockout MDA-MB-231 cells in Nude mice (*n* = 5 per group). **f** The number of metastatic nodules in the lung. **g** pathologic analysis of lung tissues by HE staining. **h** Tumour area ratio of lung metastatic. Scale bar: 500 μm. The data shown as mean ± SD and represent 3 independent experiments, and *p < 0.05; **p < 0.01.

In wound healing assays, autophagy inhibition by ATG7 KO in TNBC cells led to a significantly decreased migration rate compared with that of control WT cells, and FRAX486 treatment under autophagy inhibition conditions did not further decrease TNBC migratory ability (Fig. 2a, b). Consistently, autophagy inhibition also decreased the invasion rate of TNBC cells, and FRAX486 could not further inhibit the invasion of ATG7 KO TNBC cells (Fig. 2c, d). The metastatic ability of TNBC MDA-MB-231 cells in vivo was abolished by ATG7 KO (Figs. 1e, f and 2e, f). FRAX486 treatment under autophagy inhibition conditions did not further suppress the lung metastasis of TNBC cells (Fig. 2e–h and Supplementary Fig. S4a, b).

These data demonstrated that FRAX486 suppresses TNBC invasion and metastasis via autophagy inhibition.

### FRAX486 inhibits autophagy in TNBC cells

**Fig. 3:**
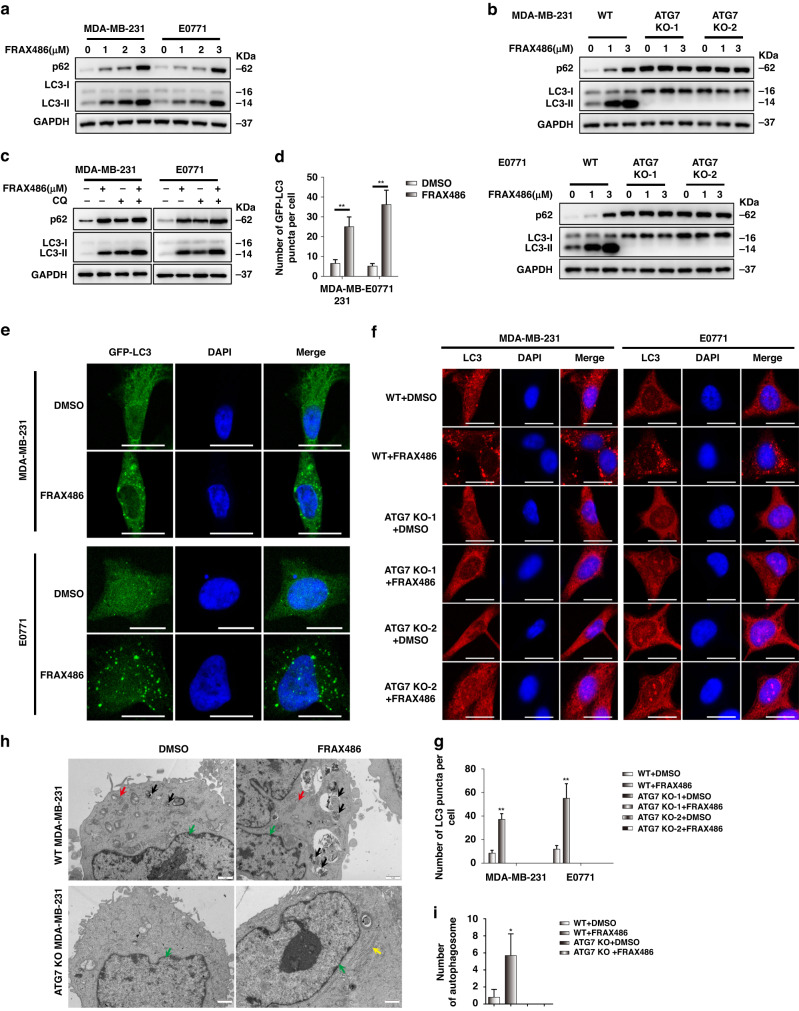
FRAX486 inhibits autophagy in TNBC cells. **a**, **b** Immunoblot analysis of LC3 and p62 protein levels in WT or ATG knockout TNBC cells treated with the indicated concentrations of FRAX486 for 24 h. **c** Immunoblot analysis of LC3 and p62 protein levels in WT TNBC cells treated with FRAX486 and/or CQ for 24 h. **d**, **e** Formation of exogenous LC3 puncta in TBNC cells treated with 2 μM FRAX486 for 24 h. Scale bar: 20 μm. **f**, **g** Immunofluorescence analysis of endogenous LC3 puncta in WT and ATG7 knockout TBNC cells treated with 2 μM FRAX486 for 24 h. Scale bar: 20 μm. **h**, **i** Transmission electron microscopy detected the autophagosomes in WT and ATG7 knockout TBNC cells. Scale bar: 1 μm. Red arrow: mitochondria; black arrow: autolysosome; yellow arrow: endoplasmic reticulum (ER); green arrow: nucleus. The data shown as mean ± SD and represent 3 independent experiments, and *p < 0.05; **p < 0.01.

We then tried to confirm the inhibition of autophagy in TNBC cells by FRAX486. The results revealed that FRAX486 increased the protein levels of the autophagy marker proteins p62 and LC3-II in TNBC cells (Fig. 3a), which is a hallmark of autophagy inhibition. In addition, FRAX486 had a limited impact on p62 and LC3-II levels in ATG7 KO cells (Fig. 3b), indicating that FRAX486 increased the protein levels of p62 and LC3-II through inhibition of autophagy. Furthermore, FRAX486 treatment promoted the accumulation of p62 and increased the ratio of LC3-II and LC3-I but only slightly when autophagy was already blocked by CQ treatment (Fig. 3c), indicating the function of FRAX486 in the inhibition of autophagy. We then analysed autophagic flux by fluorescence imaging of autophagosomes, as shown by LC3 puncta (Supplementary Fig. S5a, b). We observed an obvious accumulation of GFP-LC3 puncta in TNBC cells treated with FRAX486 (Fig. 3d, e). Furthermore, blockage of autophagosome formation by ATG7 KO completely abolished the accumulation of LC3 puncta induced by FRAX486 (Fig. 3f, g), indicating that the increase in autophagosomes caused by FRAX486 was mediated by inhibition of autophagosome–lysosome fusion and degradation. Final verification with transmission electron microscopy (TEM) confirmed the accumulation of mature double-layered autophagosomes in FRAX486-treated cells (Fig. 3h, i).

In summary, these results confirmed the inhibition of autophagy by FRAX486.

### FRAX486 inhibits autophagosome–lysosome fusion by promoting STX17 degradation

**Fig. 4:**
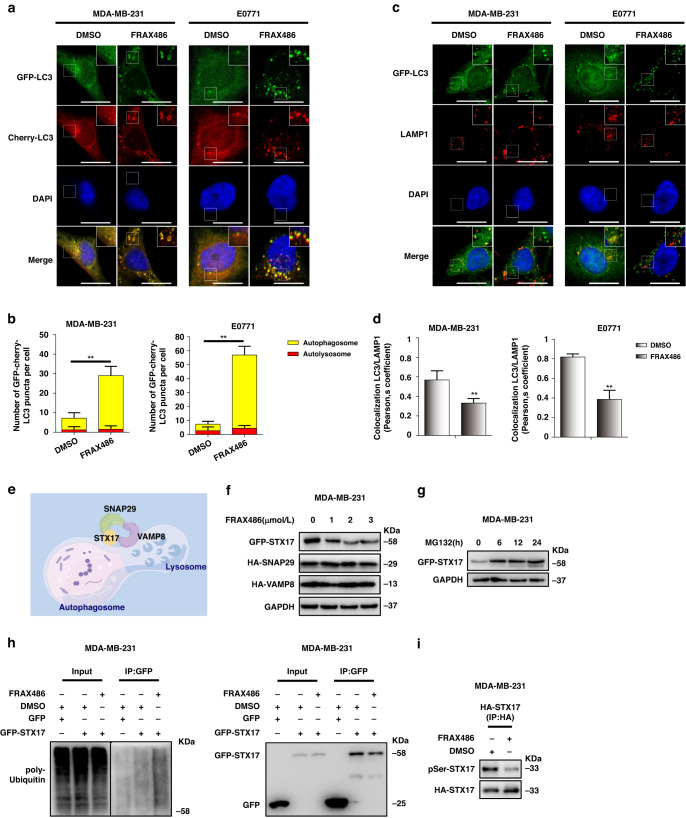
FRAX486 inhibits autophagosome–lysosome fusion via STX17 degradation. **a**, **b** Fluorescence microscopy detected the autophagy flux in cells with stable expression of mCherry-GFP-LC3 treated with 2 μM FRAX486. Scale bar: 20 μm. **c**, **d** Immunofluorescence analysis of co-localisation of GFP-LC3 and LAMP1 in cells treated with 2 μM FRAX486. Scale bar: 20 μm. **e** Schematic diagram of the role of the STX17-SNAP29-VAMP8 complex in autophagosome–lysosome fusion. **f** Immunoblot determined the protein levels of STX17, SNAP29 and VAMP8 in MDA-MB-231 cells treated with the indicated concentrations of FRAX486 for 24 h. **g** The effect of MG132 on STX17 degradation in MDA-MB-231 cells. **h** MDA-MB-231 cells with GFP or GFP-STX17 transfection were submitted to FRAX486 treatment or not. Immunoprecipitation (IP) with anti-GFP was used to detect STX17 poly-ubiquitin. **i** MDA-MB-231 cells with HA -STX17 transfection were submitted to FRAX486 treatment or not. Immunoprecipitation (IP) with anti-HA was used to detect STX17 phosphorylation. The data shown as mean ± SD and represent 3 independent experiments, and *p < 0.05; **p < 0.01.

Autophagy is a complex multistep process that consists of autophagosome initiation, extension, autophagosome–lysosome fusion and degradation [16]. The above results show the inhibition of autophagy by FRAX486. We next assessed the molecular mechanism of FRAX486-induced autophagy inhibition. mCherry-GFP-LC3 autophagic flux analysis was performed, and the results showed that FRAX486 promoted the accumulation of yellow puncta (autophagosomes) but had a limited impact on red puncta (autolysosomes) (Fig. 4a, b). The accumulation of autophagosomes but not autolysosomes is supposed to be the result of dysregulation of fusion signals or lysosomal acidity [29]. LysoTracker staining revealed that FRAX486 had no impact on lysosomal acidity, as evidenced by similar fluorescent intensities in control and FRAX486-treated cells (Supplementary Fig. S6a, b). Previous studies [30, 31] have shown that lysosomal exocytosis is involved in cancer cell invasion. To determine whether FRAX486 affects the intracellular distribution of lysosomes, the lysosome marker lysosome-associated membrane protein-1 (LAMP1) was covisualized with the cytoskeleton marker actin. LAMP1-positive vesicles did not show cell periphery relocalization (Supplementary Fig. S6c), ruling out the possibility that FRAX486 affects the intracellular distribution of lysosomes. These results suggested that FRAX486 inhibits the fusion of autophagosomes with lysosomes. Therefore, we analysed the colocalization of the autophagosome marker GFP-LC3 and the lysosome marker LAMP1 to analyse the fusion of autophagosomes and lysosomes. The results showed that FRAX486 caused downregulation of LC3-LAMP1 colocalization (shown by Person’s coefficient) compared with that in control cells (Fig. 4c, d). Thus, FRAX486 likely induces its inhibitory effect by dysregulating autophagosome–lysosome fusion. The SNARE complex, which consists of STX17, SNAP29 and VAMP8, plays a crucial role in autophagosome–lysosome fusion (Fig. 4e) [32]. Therefore, we measured the protein levels of these SNARE factors and found that FRAX486 specifically reduced the protein levels of STX17 in MDA-MB-231 cells (Fig. 4f). As reported [33], STX17 is degraded by the proteasome, which was evidenced by the upregulation of STX17 protein levels by MG132 treatment (Fig. 4g). Furthermore, the results showed that FRAX486 induced the ubiquitination of STX17 in MDA-MB-231 cells (Fig. 4h). Given that FRAX486 is a PAK kinase inhibitor [22], we examined the effect of FRAX486 on the phosphorylation of STX17. The results showed that FRAX486 suppressed the phosphorylation of STX17 (Fig. 4i). Studies have shown that protein phosphorylation can reduce ubiquitination and thus protect proteins from proteasomal degradation [34, 35]. Thus, we hypothesised that FRAX486 induces the ubiquitination and proteasomal degradation of STX17 by inhibiting the phosphorylation of STX17, which suppresses assembly of the SNARE complex and consequently blocks autophagosome–lysosome fusion.

### FRAX486 targets PAK2 to induce STX17 degradation

**Fig. 5:**
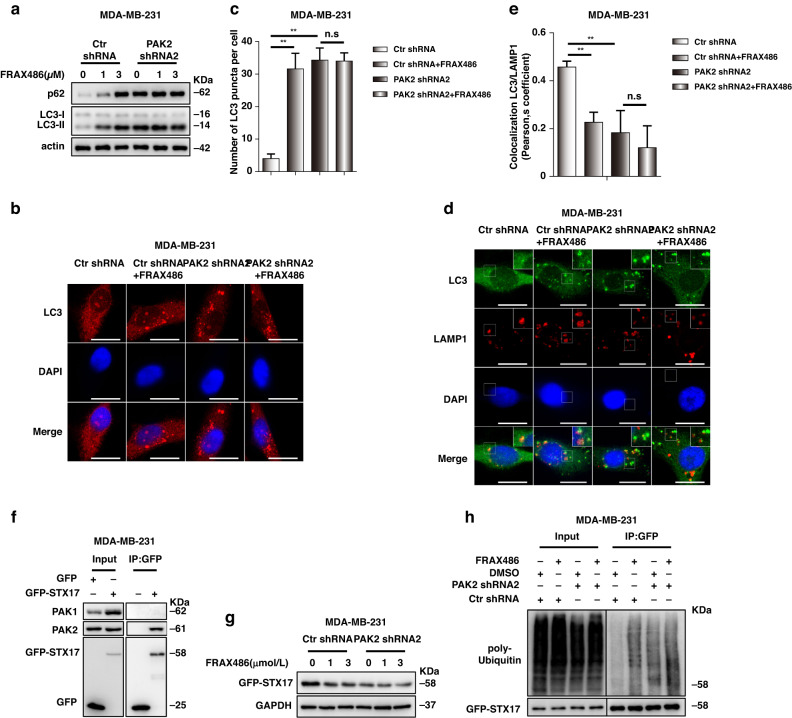
FRAX486 targets PAK2 to induce STX17 degradation. **a** MDA-MB-231 cells with ctr shRNA or PAK2 shRNA transfection were subjected to the indicated concentrations of FRAX486. Immunoblot analysis of LC3 and p62 protein levels. **b**, **c** Immunofluorescence analysis of endogenous LC3 puncta in ctr shRNA and PAK2 shRNA MDA-MB-231 cells treated with 2 μM FRAX486 or not. Scale bar: 20 μm. **d**, **e** Immunofluorescence tested co-localisation of LC3 and LAMP1 in cells treated as in (**b**). Scale bar: 20 μm. **f** MDA-MB-231 cells with GFP or GFP-STX17 transfection were used to investigate the interaction of STX17 with PAK1 and PAK2. **g** Immunoblot analysis of GFP-STX17 protein levels in cells treated as in (**a**). **h** MDA-MB-231 cells with Ctr shRNA or PAK2 shRNA transfection were submitted to 2 μM FRAX486 treatment or not. Immunoprecipitation (IP) with anti-GFP was used to detect STX17 poly-ubiquitin. The data shown as mean ± SD and represent 3 independent experiments, and ∗*p* < 0.05; ∗∗*p* < 0.01.

As reported, Type I PAKs, including PAK1, PAK2 and PAK3, are known targets of FRAX486 [22, 36]. We explored the TCGA-BRAC dataset and found that PAK3 was highly expressed in normal mammary tissues (Supplementary Fig. S7a), indicating that FRAX486 inhibition of TNBC cells did not occur through targeting PAK3. Thus, PAK1 and PAK2 were considered potential targets of FRAX486 in TNBC cells. Knockdown of PAK2 instead of PAK1 significantly increased the protein levels of p62 and LC3-II in MDA-MB-231 cells (Supplementary Fig. S7b), which was consistent with FRAX486 treatment, suggesting that PAK2 is the target subject to FRAX486 inhibition. Thus, the role of PAK2 in FRAX486-induced autophagy inhibition was further investigated. The results revealed that p62 protein levels were not further increased by FRAX486 treatment in PAK2 knockdown cells (Fig. 5a). Fluorescent imaging confirmed that FRAX486 could not further block autophagic flux in PAK2 knockdown cells, as shown by the consistent LC3 puncta numbers in PAK2 knockdown and PAK2 knockdown plus FRAX486-treated cells (Fig. 5b, c). Moreover, FRAX486 treatment or PAK2 knockdown inhibited the colocalization of LC3 and LAMP1, but PAK2 knockdown plus FRAX486 treatment showed no overlay effects (Fig. 5d, e), indicating that FRAX486 inhibited autophagosome–lysosome fusion by targeting PAK2. Co-IP results showed that PAK2, but not PAK1, interacted with STX17 in MDA-MB-231 cells (Fig. 5f). PAK2 knockdown in MDA-MB-231 cells decreased STX17 protein levels, which could not be further decreased by FRAX486 treatment (Fig. 5g). Furthermore, PAK2 knockdown facilitated the ubiquitination of STX17, which was not further increased by FRAX486 treatment in PAK2 knockdown cells (Fig. 5h). These data indicated that FRAX486 promotes STX17 ubiquitination and degradation through PAK2 inhibition.

Together, the above results revealed that FRAX486 targets PAK2 and promotes STX17 degradation to inhibit autophagy.

### FRAX486 targets PAK2 to inhibit TNBC migration and metastasis

**Fig. 6:**
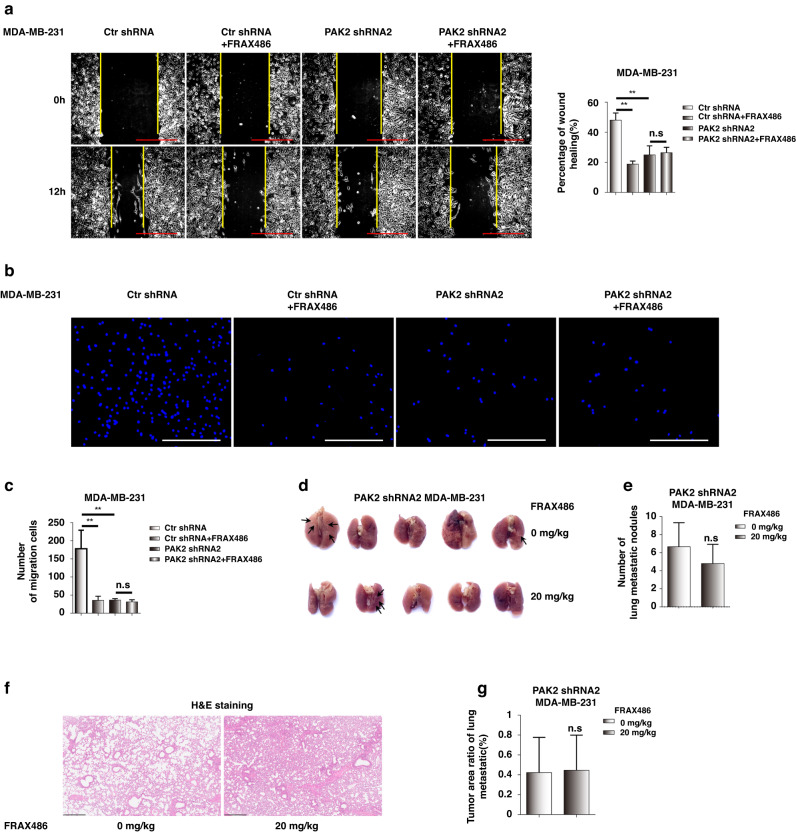
FRAX486 targets PAK2 to inhibit TNBC migration and metastasis. **a** Wound healing assay detected the wound healing rate in MDA-MB-231 cells with control shRNA or PAK2 shRNA transfection were subjected to the 2 μmol/L FRAX486 or not. Scale bar: 500 μm. **b**, **c** Migration assay analysis of number of migration cells in cells treated as in (**a**). Scale bar: 500 μm. **d** Lung metastasis of PAK2 knockout MDA-MB-231 cells in Nude mice (*n* = 5 per group). **e** The number of metastatic nodules in the lung. **f** Pathologic analysis of lung tissues by HE staining. **g** Tumour area ratio of lung metastatic. Scale bar: 500 μm. The data shown as mean ± SD and represent 3 independent experiments, and *p < 0.05; **p < 0.01.

Finally, we investigated the role of PAK2 in TNBC migration and metastasis and assessed whether the effects of FRAX486 on TNBC cells were mediated by targeting of PAK2. Wound healing assays showed that PAK2 knockdown inhibited the migration of MDA-MB-231 cells, but FRAX486 treatment did not exert further effects on PAK2 knockdown cells (Fig. 6a). The invasion of TNBC cells was also abolished by PAK2 knockdown (Fig. 6b, c). The lung metastatic ability of MDA-MB-231 cells in vivo was abolished in the PAK2 knockdown group compared with the control group, as shown in Fig. 1e (Fig. 6d, e). More importantly, FRAX486 treatment did not further reduce the lung metastasis capacity of PAK2 knockdown MDA-MB-231 cells (Fig. 6d, e and Supplementary Fig. S8a). Metastatic nodules were seldom identified in PAK2 knockdown group mice by HE staining, and FRAX486 treatment did not exert further effects (Fig. 6f, g and Supplementary Fig. S8b).

These results demonstrated that PAK2 is the target of FRAX486 in the inhibition of TNBC migration and metastasis in vitro and in vivo.

## Discussion

In this study, we verified the impacts and underlying mechanism of the PAK2 inhibitor FRAX486 on TNBC. We found that FRAX486 represses TNBC invasion and metastasis through autophagy inhibition both in vitro and in vivo. FRAX486 inhibits autophagy by blocking autophagosome–lysosome fusion, which is mediated by PAK2 inhibition-induced degradation of STX17.

Cancer metastasis is a multistep process that includes cancer cell invasion, haematogenous dissemination, vessel invasion, localisation in distal organs and growth [37, 38]. A growing body of evidence indicates that the EMT process is crucial for TNBC metastasis [39]. In our study, FRAX486 inhibited TNBC invasion in wound healing and migration assays and metastasis in mouse models. Notably, high protein levels of the epithelial marker E-cadherin are a hallmark of EMT inhibition [40, 41]. Recently, it was reported that autophagic degradation of E-cadherin promotes the EMT process in several types of cancers, such as lung squamous cell carcinoma, melanoma, and hepatocellular cancer [26–28]. We found that FRAX486 increased the protein level of E-cadherin in wild-type TNBC cells but not in ATG7 KO cells, indicating a crucial role of autophagy in FRAX486-induced E-cadherin upregulation. Usually, intracellular protein degradation is mediated by the proteasome or autophagy‒lysosome pathway [42–44]. In our study, we found that E-cadherin protein expression was maintained when FRAX486 was applied, as this drug-induced autophagy inhibition. These results also confirmed the relationship between the EMT process and the autophagy pathway.

After we identified the crucial role of autophagy in EMT suppression mediated by FRAX486, the molecular mechanism of autophagy inhibition was investigated. Type I PAKs, which include PAK1, PAK2 and PAK3, are the targets of FRAX486 [22, 36]. In TNBC cells, FRAX486 was verified to target and inhibit PAK2 in our study. PAK2 plays important roles in embryogenesis and in the aetiology of autism [45, 46]. In addition, PAK2 regulates cell survival [47, 48]. However, the impact of PAK2 on autophagy has seldom been investigated. In our study, PAK2 knockdown increased the protein levels of the autophagic markers p62 and LC3, indicating the blockage of autophagic flux. Furthermore, PAK2 knockdown abolished the inhibitory effect of FRAX486 on autophagy. These results highlight that PAK2 is a positive modulator of autophagy and that FRAX486 inhibits autophagy by targeting PAK2. As a downstream signal, the SNARE complex [49], consisting of STX17, VAMP8 and SNP29, was assessed for its ability to inhibit autophagosome–lysosome fusion upon FRAX486 treatment. STX17 localises to autophagosomes and recruits SNP29 to form the STX17-SNP29 complex. Then, SNP29 interacts with VAMP8, which is located on lysosomes, to form the SNARE complex and mediate autophagosome–lysosome fusion [32, 50]. In our study, FRAX486 induced the ubiquitination and degradation of STX17 and consequently blocked autophagy. Mechanistically, PAK2 interacts with STX17, which is supposed to protect STX17 from proteasome-mediated degradation. Therefore, FRAX486 inhibits PAK2 expression and promotes ubiquitination and eventual degradation of STX17. Thus, autophagy is blocked at the fusion stage because of the dysfunction of the SNARE complex.

In summary, our study showed that FRAX486 is a dual EMT/autophagy inhibitor in TNBC cells. Autophagy inhibition by FRAX486 increases E-cadherin protein levels and consequently suppresses EMT and metastasis in TNBC cells. FRAX486 targets PAK2 and promotes ubiquitination and proteasome-mediated degradation of STX17, which leads to dysfunction of the SNARE complex and subsequent suppression of autophagosome–lysosome fusion. Our study highlighted the essential role of the PAK2-STX17 axis in autophagy modulation in TNBC cells and suggested that FRAX486 is a potential treatment for metastatic TNBC.

## Materials and methods

### Cell culture and reagents

HEK293T, MDA-MB-231 and MCF-10A were purchased from National Infrastructure of Cell Line Resource (Beijing, China). E0771 cell line was kindly gifted from Prof. Bisen Ding (Sichuan University). HEK293T and MDA-MB-231 were maintained in Dulbecco’s modified Eagle’s medium (DMEM) supplemented with 10% foetal bovine serum (FBS) and 1% penicillin–streptomycin (P-S). E0771 was cultured in RPMI-1640 medium supplemented with 10% FBS and 1% P-S. MCF-10A was maintained in DMEM/F12 medium supplemented with 5% horse serum, 20 ng/mL epidermal growth factor (EGF) and 1% P-S. All cell lines were cultured under 5% CO_2_ atmosphere at 37 °C.

### CCK-8 assays

CCK-8 assays were performed in 96-well plates, MDA-MB-231 and E0771 cells were seeded in 96-well plates at cell density of 3 × 10^4^/well. After exposing to certain concentrations of FRAX486 (HY-15542B, MCE), cells were incubated with Cell Counting Kit-8 reagent (CCK-8, Target Molecule Corporation, Boston) according to the manufacturer’s instruction at scheduled time points, and quantified by measuring absorbance at 450 nm. Three independent experiments were performed.

### Would healing assays

WT or ATG7 KO MDA-MB-231 and WT or ATG7 KO E0771 cells were seeded in 6-well plates at density of 2 × 10^6^/well for overnight incubation. Cells were scratched and wash with phosphate-buffered saline (PBS) for three times. Next, cells were maintained in DMEM supplemented with FRAX486 (0 and 2 μM) and 1% P-S for 24 h. Marked scratched areas were captured by inverted microscope (Carl Zeiss) at 0, 12 and 24 h. Results were analysed by Fiji software (https://fiji.sc/).

### Migration assays

WT or ATG7 KO MDA-MB-231 and WT or ATG7 KO E0771 cells were seeded in 6-well plates at density of 3 × 10^5^/well, and treated with FRAX486 (0 and 2 μM) for 24 h. Control and treated cells were digested and seeded 1 × 10^4^/well in the Transwell (Transwell BD Matrigel, 3422, Costar) inserts in DMEM medium, and placed in 24-well plates, which were infused with 500 μL DMEM or RPMI-1640 medium supplemented with 10% FBS and 1% P-S. Equivalent medium served as control. After incubation for 48 h, the inner surface of transwell inserts were scratched and outer surface were fixed by 4% paraformaldehyde and stained with DAPI. Images were captured and analysed by microscope.

### Western blotting

Total proteins were extracted from cells by Hu Buffer, which was consisted by 48% urea, 5% sodium dodecyl sulfate (SDS), 1.5% dithiothreitol (DTT), 0.1% bromophenol blue and 20% 1 M Tris-HCl (v/v). Total proteins were separated by 8–15% polyacrylamide gel electrophoresis and analysed by Western blot. The bands were photographed by ChemiDoc MP Imaging system (Bio-Rad) and quantified by Fiji software. Antibodies used in western blotting are listed in Supplementary Table 1.

### Transfection, knockdown and knockout

Screening of stably transfected cell lines using a lentiviral system. As previously reported, lentiviral vectors encoding the human STX17, VAMP8 and SNAP29 genes, and PAK1, PAK2 shRNA or control shRNA were infected into TBNC cells. After 48 h, infected cells were selected for 2 weeks with 5 μg/mL puromycin (A610593-0025, BBI) and stable cell lines were established. We chose two different shRNAs against each target gene (PAK2, PAK1). Ctr shRNA: 5′-GCGCGATAGCGCTAATAATTT-3′; PAK1 shRNA1: 5′-GCATTCGAACCAGGTCATTCA-3′; PAK1 shRNA2: 5′-CCCTAAACCATGGTTCTAAAC-3′; PAK2 shRNA1: 5′-CGGGATTTCTTAAATCGATGT-3′; PAK2 shRNA2: 5′-GACAGGAGGTTGCTATCAAAC-3′.

### Immunofluorescence staining and confocal microscopic analyses

To analyse the autophagic status, MDA-MB-231 and E0771 cells were infected by lentivirus to construct stable overexpression cell lines, which expressed GFP-LC3 and mCherry-GFP-LC3. Then, cells were treated with FRAX486 (2 μM) for 24 h, and fixed with 4% paraformaldehyde (PFA) for 10 min, stained with DAPI for 15 min. Images were captured and analysed by confocal microscope (Carl Zeiss).

For immunofluorescence staining (IF), stable GFP-LC3 overexpression MDA-MB-231 and E0771 cells were also treated with FRAX486 (2 μM) for 24 h and fixed with 4% PFA for 10 min. Next, cells were permeabilized with 0.2% Triton X-100 for 10 min, blocked with 5% FBS in PBS for 30 min, and incubated with anti-LAMP1 antibody overnight at 4 °C. Then, cells were incubated with Alexa Fluor594-conjugated second antibody for 1 h, and stained with DAPI for 15 min. Images were also captured and analysed by confocal microscope.

Additionally, to verified the lysosomal acidity, MDA-MB-231 and E0771 cells were treated with FRAX486 for 24 h, stained with LysoTracker Red (Beyotime) for 30 min, and analysed by microscope.

### Transmission electron microscopy

Transmission electron microscopy (TEM) was adopted to visualise the autophagosome and autolysosome via routine protocols. Briefly, MDA-MB-231 cells were treated with vehicle (DMSO) and FRAX486 (2 μM) for 24 h. Cells were fixed in 3% glutaraldehyde phosphate buffer and 1% osmium tetroxide. Then, samples were dehydrated in different concentrations of acetone, embedded, and sliced (50 nm). Slices were dyed, stained with 4% uranium acetate for 10 min, and stained with lead citrate for 2 min. Next, slices were visualised and captured by transmission electron microscope (JEM-1400 FLASH, JEOL, Japan).

### Co-immunoprecipitation

MDA-MB-231 cells stably expressing GFP-STX17, control shRNA and PAK2 shRNA2 were seeded in 10-cm petri dishes, and were treated with vehicle (DMSO) and FRAX486 (2 μM) for 24 h. Cells were harvested and lysed by pre-cooling lysis buffer (0.8775% NaCl, 5% glycerine (v/v), 2.5% 1 M Tris-Hcl (v/v, pH7.4), 0.5% Triton x-100 (v/v)) for 30 min. After centrifuge at 500 rpm for 5 min, supernatant was reserved as input and incubated with GFP- or HA-coated beads at 4 °C for 2 h. Targeted proteins (or complex) were precipitated by centrifugation (1500 rpm for 3 min) and washed with wash buffer (lysis buffer containing 1% NP40) for 5 times. Proteins were eluted by Hu buffer and heated at 65 °C for 20 min. Results were analysed by western blotting.

### Quantitative real-time PCR

Total RNA was extracted from MDA-MB-231 and E0771 by FastPure Cell/Tissue Total RNA Isolation Kit (Vazyme, Nanjing, China) according to the manufacturer’s protocol. RNA samples were assessed by NanoDrop ND-1000 spectrophotometer (NanoDrop Technologies). cDNA was synthesised from 500 ng of RNA using HiScript III Enzyme Mix (Vazyme). RT-PCR mixtures were prepared by 2 × SYBR qPCR Master Mix (Vazyme) according to the manufacturer’s protocol using the following procedure: 95 °C for 30 s, followed by 40 cycles of 95 °C for 10 s and 60 °C for 30 s, and final 72 °C for 10 min. Experiments were carried out in triplicate. GAPDH was served as internal control, all primers sequences were: human GAPDH, forward CACATCGCTCAGACACCATG and reverse TGACGGTGCCATGGAATTTG; human CDH1, forward CCCGGGACAACGTTTATTAC and reverse GCTGGCTCAAGTCAAAGTCC; mouse GAPDH, forward TTCACCACCATGGAGAAGGC and reverse GGCATGGACTGTGGTCATGA; mouse CDH1, forward CTCCAGTCATAGGGAGCTGTC and reverse TCTTCTGAGACCTGGGTACAC.

### Public data analysis

To assess the expressions of PAKs in BC and normal mammary tissues, we used RTCGA R package to download and retrieve gene expression matrix and clinical information of TCGA-BRCA dataset. Differential analysis between groups was calculated by Limma package (v3.50.5) by using the linear model. adj.*p*.val < 0.05 was regarded as significant. Results were visualised by pheatmap package.

### Animal models

Thirty-six-week-old female Balb/c nude mice were purchased from Gempharmatech Co., Ltd (Nanjing, China) and kept in specific pathogen-free (SPF) condition. After one week, mice were randomly assigned to three groups (*n* = 10), and injected with 100 μL cell suspension which contained 2 × 10^6^ WT, ATG7 KO, PAK2 shRNA2 MDA-MB-231 cells, via caudal vein, respectively. Five days after injection, these groups were further divided into two subgroup each (*n* = 5), and treated with vehicle and 20 mg/kg FRAX486 by gavage for 5 days each week. After 7 weeks’ treatment, mice were euthanized by CO2 asphyxiation, and lung tissues were collected, and metastatic nodules were quantified. Lung tissues were then reserved for further pathologic analysis. The animal experiments were approved by the Biomedical Research Ethics Committee of West China Hospital of Sichuan University.

### Hematoxylin-eosin and immunohistochemical staining

Tissue samples obtained from animal models were fixed in 10% formalin and embedded in paraffin according to routine procedures. Antigen retrieval was performed by boiling in citrate-based buffer (pH 6.0) after blocking endogenous peroxidase activity. Sections were incubated with primary antibodies overnight followed by 1 h of second antibody incubation. Staining was visualised by using a DAB staining Kit (Gene Tech), and scanned with Pannoramic Scanner (3DHISTECH, Budapest, Hungary). Hematoxylin-eosin (HE) staining followed the standard protocol. Briefly, Embedded tissues were sectioned at an interval of 4 μm, and stained with hematoxylin aqueous solution for 10 min after routine dewaxing. The slices were separated in acid water and ammonia water, and rinsed with water for 1 h. Then, slices were dehydrated and covered with glass for observation.

### Statistical analysis

All resulting data were statistically analysed using a two-tailed Student’s *t*-test. The significance was denoted as *p < 0.05; **p < 0.01.

### Supplementary information

Supplementary--Lyv et al.
