# Supplementary material for: PAK inhibitor FRAX486 decreases the metastatic potential of triple-negative breast cancer cells by blocking autophagy
Source: Br J Cancer. 2023 Dec 18;130(3):394–405. doi: 10.1038/s41416-023-02523-4 (PMC10844298; doi:10.1038/s41416-023-02523-4)
Supplement: Supplementary file 1 — Supplementary--Lyv et al. [file 41416_2023_2523_MOESM1_ESM.docx]

**PAK Inhibitor FRAX486 Decreases the Metastatic Potential of Triple-Negative Breast Cancer Cells by Blocking Autophagy**

Liang Lyu^1,#^, Haiyan Li^1,#^, Kefeng Lu^1^, Shu Jiang^1^ and Huihui Li^1,2,*^

^1^Department of Neurosurgery, State Key Laboratory of Biotherapy, West China Hospital, Sichuan University, Chengdu 610041, China

^2^West China Second University Hospital, Sichuan University, Chengdu 610041, China

^#^These authors contributed equally to this work

^*^Correspondence: Huihui Li ([lihuihui@scu.edu.cn](mailto:lihuihui@scu.edu.cn)), ORCID: 0000-0001-6004-3086

**Supplementary Figure S1**

**
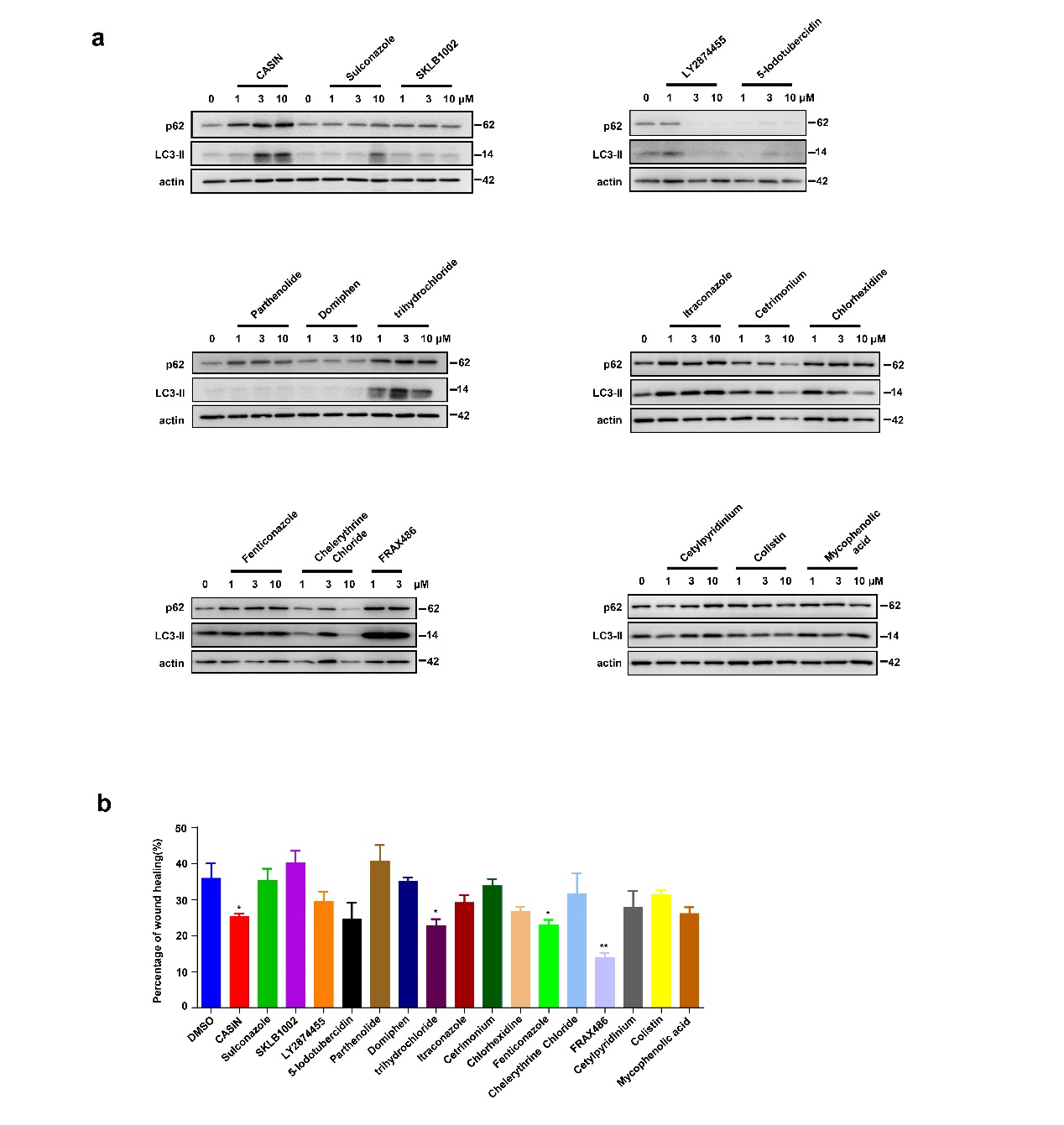
Figure S1 a,** Immunoblot analysis of LC3 and p62 protein levels in MDA-MB-231 cells treated with the indicated concentrations of 17 small molecule chemicals for 24 hours. **b,** Wound healing assay detected the migration MDA-MB-231 cells treated with 17 small molecule chemicals. The data shown as mean ± SD and represent 3 independent experiments, and *p < 0.05; **p < 0.01.

**Supplementary Figure S2**

**
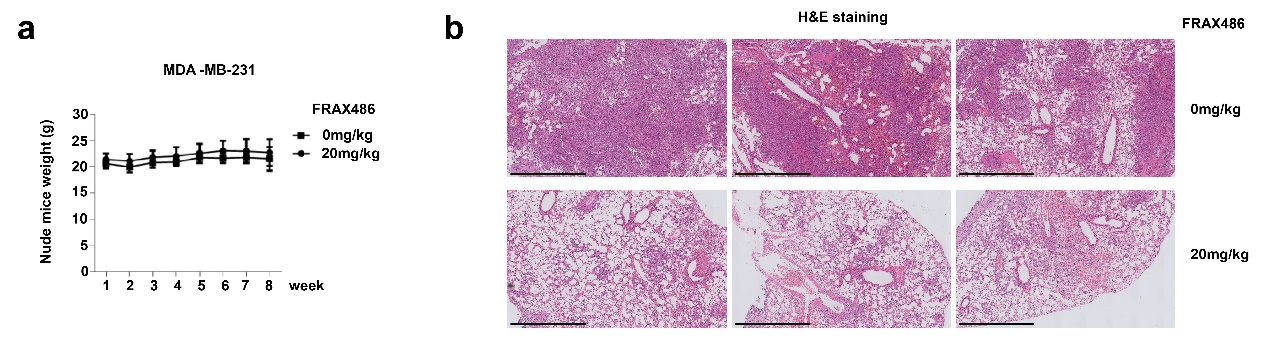
**

**Figure S2 a,** Body weight of Nude mice injected with 2×10^6^ MDA-MB-231 cells and treated with 20mg/kg FRAX486 or not. **b,** Pathologic analysis of lung tissues by HE staining. Scale bar: 500 μm.

**Supplementary Figure S3**

**
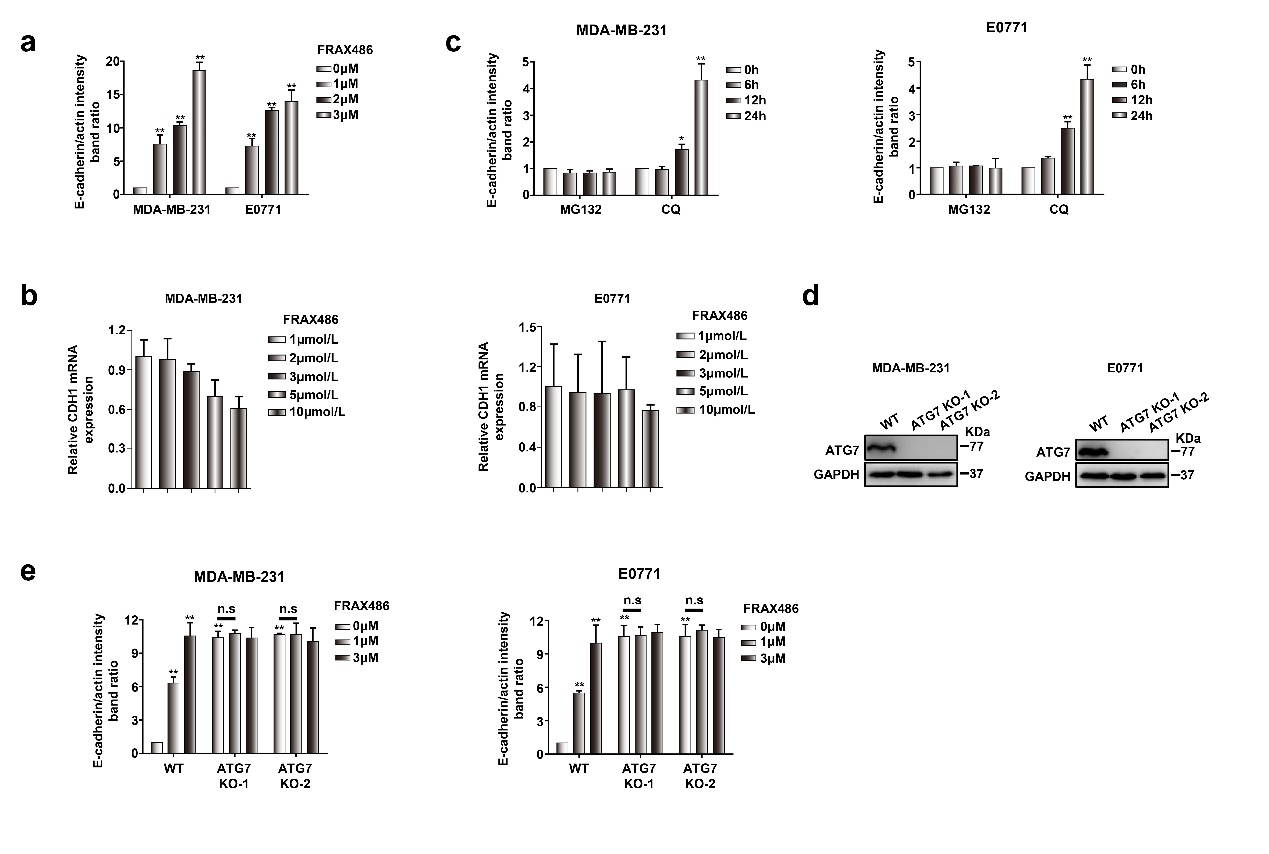
**

**Figure S3 a,** Semi-quantitative analysis of E-cadherin the expression levels presented in Fig. 1i. **b,** Expression of CDH1 was examined by qPCR after FRAX486 treatment. **c,** The relative expression of E-cadherin in Fig. 1j. **d,** Immunoblots analysis of ATG7 protein levels in WT and ATG7 KO TNBC cells. **e,** Semi-quantitative analysis of E-cadherin the expression levels presented in Fig. 1k. The data shown as mean ± SD and represent 3 independent experiments, and ∗p < 0.05; ∗∗p < 0.01 compared with DMSO or 0 hour treatment only.

**Supplementary Figure S4**

**
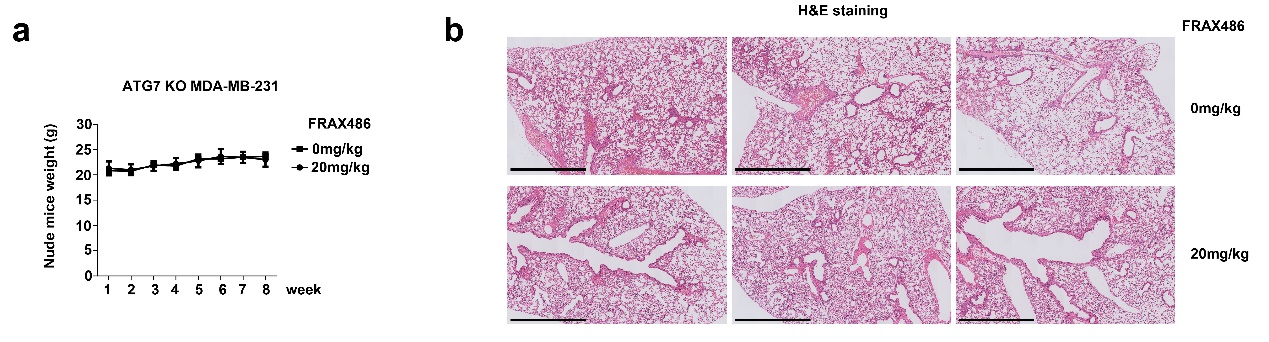
**

**Figure S4 a,** Body weight of Nude mice injected with 2×10^6^ ATG7 knock-out MDA-MB-231 cells and treated with 20mg/kg FRAX486 or not. **b,** Pathologic analysis of lung tissues by HE staining. Scale bar: 500 μm.

**Supplementary Figure S5**


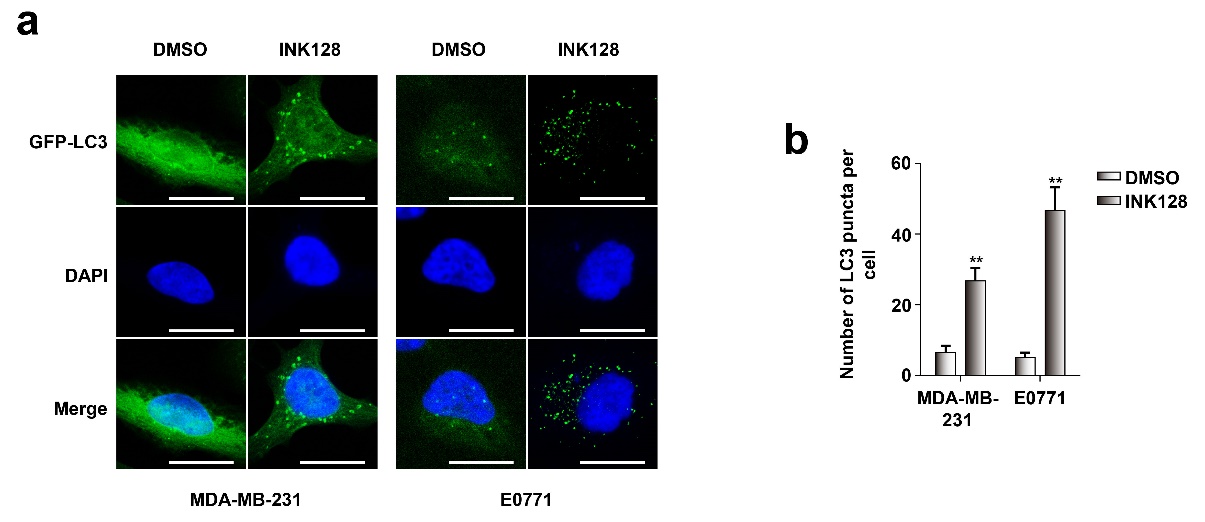


**Figure S5 a,** Formation of exogenous LC3 puncta in GFP-LC3 stable expressed TBNC cells treated with 10 μmol/L INK128 for 12 hours. Scale bar: 20 μm. **b,** Number of LC3 puncta per cell. The data shown as mean ± SD and represent 3 independent experiments, and *p < 0.05; **p < 0.01.

**Supplementary Figure S6**

**
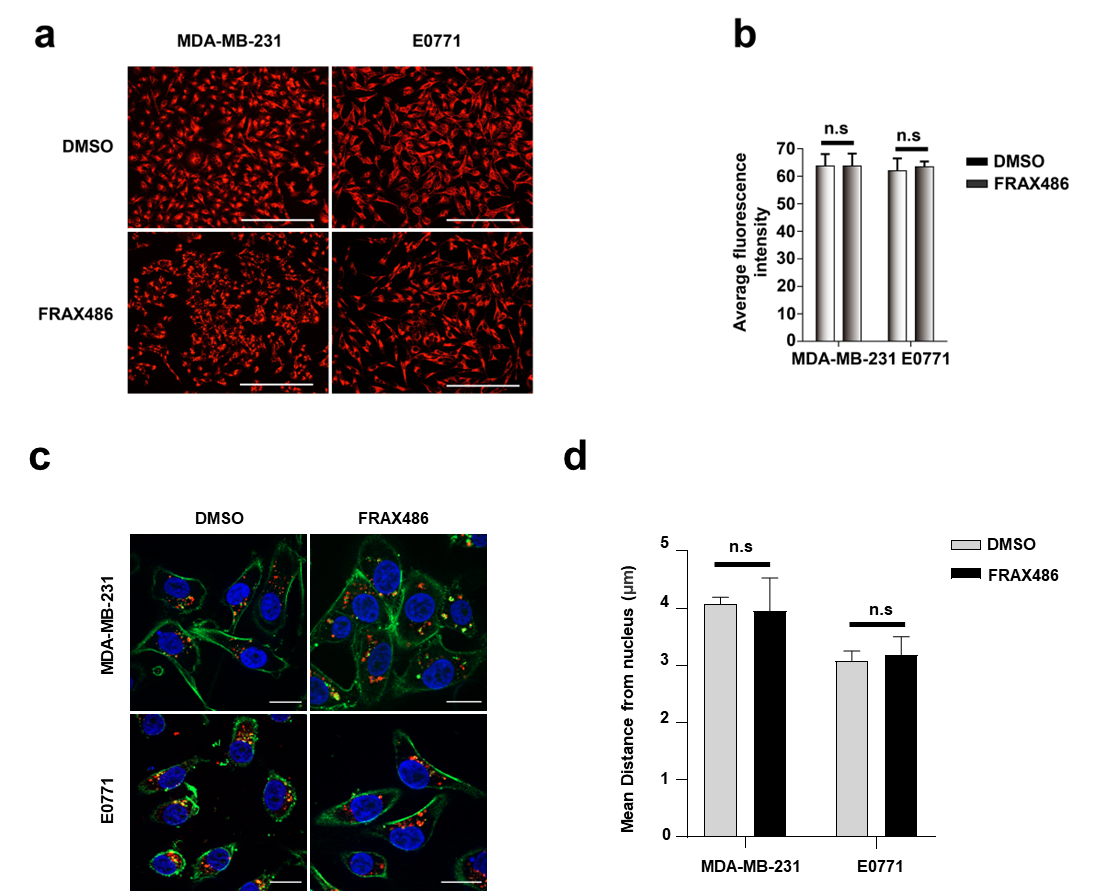
**

**Figure S6 a,** LysoTracker red staining evaluated changes in lysosomal activity in TNBC cells treated with 2 μM FRAX486. **b,** Semi-quantitative analysis of fluorescence intensity in part a. **c,** Fluorescence microscopy detected LAMP-1 (red), phalloidin (green), and DAPI (blue) in TNBC cells treated with 2 μM FRAX486. Scale bar: 20 μm. **d,** Quantification of lysosomal distribution, shown as mean distance from individual cell nuclei. The data shown as mean ± SD and represent 3 independent experiments, and *p < 0.05; **p < 0.01.

**Supplementary Figure S7**


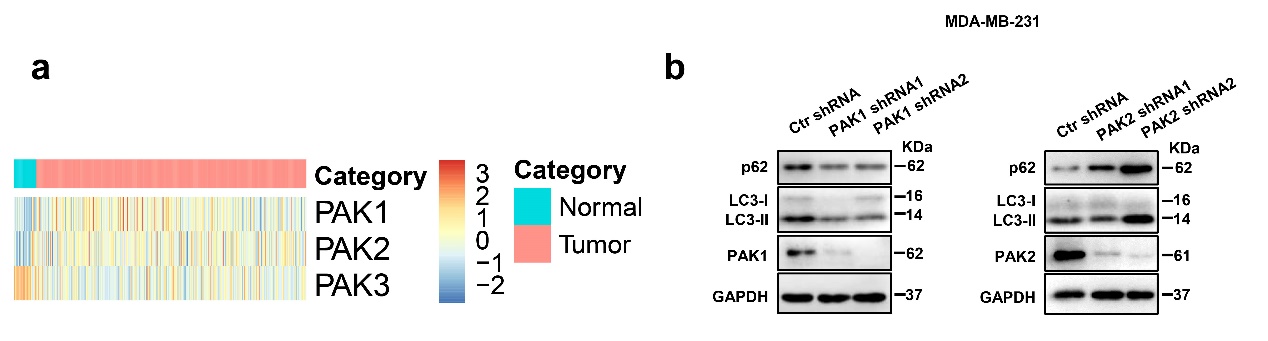


**Figure S7 a,** TCGA-BRCA database analysis of PAK1-PAK3 expression in breast cancer. **b,** Immunoblot analysis of LC3 and p62 protein levels in MDA-MB-231 cells with Ctr shRNA, PAK2 shRNA or PAK1 shRNA transfection.

**Supplementary Figure S8**


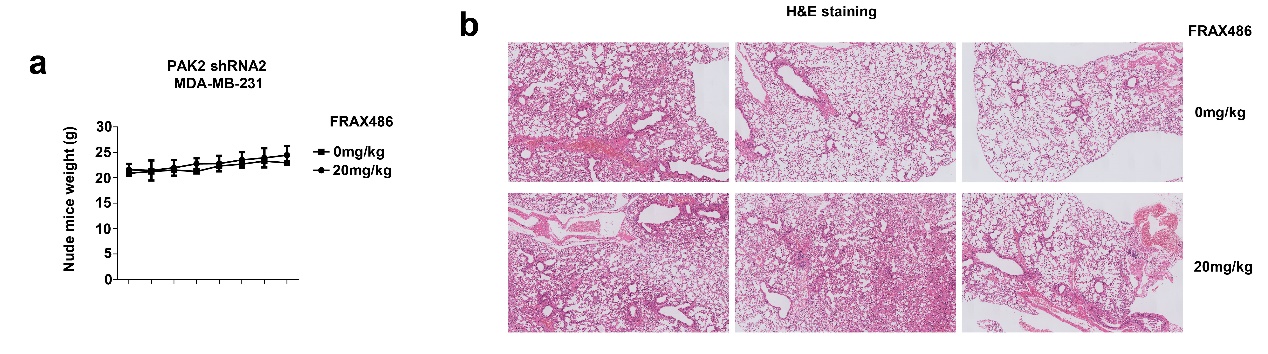


**Figure S8** **a,** Body weight of Nude mice injected with 2×10^6^ PAK2 knock-down MDA-MB-231 cells and treated with 20mg/kg FRAX486 or not. **b,** Pathologic analysis of lung tissues by HE staining. Scale bar: 500 μm.

**Supplementary Table 1**

The list of antibodies used in western blotting

| Antibody | Cat. number | Brand | Dilution ratio |
| --- | --- | --- | --- |
| GAPDH | 60004-1-Ig | proteintech | 1:10000 |
| Actin | AC026 | Abclonal | 1: 10000 |
| LC3A/B | 4108S | CST | 1: 10000 |
| p62 | ab109012 | Abcam | 1:5000 |
| GFP | sc-9996 | Santa cruz | 1:5000 |
| HA | sc-7392 | Santa cruz | 1:2000 |
| Anti-HA Affinity | 11815016001 | Roche | 25 μL |
| Alexa Fluor^TM^488 donkey Anti-Mouse IgG(H+L) | A-21202 | Thermofisher | 1:500 |
| Alexa Fluor^TM^594 donkey Anti-rabbit IgG(H+L) | A-21207 | Thermofisher | 1:500 |
| HRP-conjugated Anti-Rabbit | D-110058 | BBI | 1:5000 |
| HRP-conjugated Anti-Mouse | D-110087 | BBI | 1:5000 |
| E-Cadherin | 3195 | CST | 1:1000 |
| vimentin | ab92547 | abcam | 1:20000 |
| LAMP1 | 9091S | CST | 1:200 |
| PAK1 | 2602S | CST | 1:1000 |
| PAK2 | A4553 | abclonal | 1:1000 |
| PAK4 | 14685-1-AP | proteintech | 1:1000 |
| STX17 | 31261 | CST | 1:1000 |
| SNAP29 | Ab138500 | abcam | 1:1000 |
| VAMP8 | ab76021 | abcam | 1:5000 |
